# A systematic review of qualitative research on recently acquired HIV

**DOI:** 10.1097/QAD.0000000000003697

**Published:** 2023-08-23

**Authors:** Emily Jay Nicholls, Nicoletta Policek, Alain Volny-Anne, Bruno Spire, Fiona Burns, Elisa Ruiz-Burga, Shema Tariq

**Affiliations:** a Institute for Global Health, University College London, London; b School of Health & Society, University of Salford, Salford, UK; c Independent; d Aix Marseille Univ., Inserm, IRD, SESSTIM, ISSPAM, Marseille, France; e Royal Free London NHS Foundation Trust, London; f Mortimer Market Centre, Central and North West London NHS Foundation Trust, London, UK.

**Keywords:** behaviour, qualitative, recently acquired HIV, seroconversion, systematic review, testing

## Abstract

**Objective(s):**

Recently acquired HIV is a critical time when people may experience debilitating symptoms and is when they are most likely to pass HIV on. Qualitative research offers insights into lived experiences and a deeper understanding of the contextual factors underlying HIV acquisition. We aimed to synthesize qualitative literature on recently acquired HIV.

**Design:**

Systematic review and textual narrative synthesis.

**Methods:**

We searched MEDLINE, CINAHL Plus, PsycINFO and Sociology Database. Articles were screened, and two authors completed full text review and data extraction. Quality appraisal was conducted (Critical Appraisal Skills Programme Qualitative Studies Checklist) and certainty of findings graded (GRADE-CERQual).

**Results:**

We reviewed 1890 articles (1554 following de-duplication), excluding 1539. Fifteen articles were included and an additional article was included after updating the search. We identified 15 themes, three of which we have high confidence in: recent acquisition of HIV facilitates understanding of circumstances of HIV acquisition; indeterminate HIV tests generate uncertainty and anxiety; and people with recently acquired HIV are motivated to reduce risk of onward transmission.

**Conclusions:**

Our findings highlight the importance of continued research into recently acquired HIV, as well as the need for support to manage the emotional impact of indeterminate test results and negotiate risk reduction. We found no studies exploring sexual risk in the context of recently acquired HIV, or use of pre-exposure prophylaxis or treatment as prevention. The literature is primarily focused on HIV acquisition from an individual and behavioural perspective, neglecting important aspects of lived experience such as immediate ART, stigma, and health and wellbeing.

## Introduction

Combination HIV prevention including pre-exposure prophylaxis (PrEP), and treatment as prevention (TasP) [1,2], have reduced the incidence of HIV and HIV-associated mortality. Yet, despite these innovations, approximately 1.3 million people globally acquired HIV in 2022 [3]. While these interventions substantially reduce HIV infections, they may also influence risk perceptions, behaviours and experiences of acquiring HIV in unintended ways. Challenges remain, therefore, if we are to maximize their potential, end the HIV epidemic [4], and support those acquiring HIV to stay healthy and well.

Acute HIV infection (AHI, which we define as the first four weeks after HIV acquisition) and primary HIV infection (PHI, the six-month period after acquisition) are characterized by a surge in viraemia [5,6]. This is a critical time when people may experience debilitating symptoms and when they are most likely to pass HIV on. Qualitative studies have explored the multidimensional impacts of an HIV diagnosis [7,8]. However, experiences of recently acquired HIV may be complicated by AHI symptoms and high infectiousness, with social, behavioural and psychological impacts. Understanding experiences of recently acquired HIV can inform effective and tailored support for this population. Furthermore, people with recently acquired HIV are likely to better recall circumstances around infection, allowing us to identify and understand missed prevention opportunities.

Recently acquired HIV is well characterized clinically and epidemiologically [9–11]. However, qualitative research offers insights into experiences and deeper understanding of contextual factors underlying new HIV infections. We aimed to understand the experiences of people with recently acquired HIV by synthesizing qualitative evidence.

## Methods

We conducted a systematic review of literature on recently acquired HIV; this report follows the Preferred Reporting Items for Systematic Reviews and Meta-Analyses (PRISMA) checklist. We defined recently acquired HIV as an HIV-positive antibody test ≤12 months since a previous HIV-negative antibody test, or laboratory evidence of acute HIV infection. Articles were critically appraised, systematically evaluated and compared to generate summary findings and novel insights. We used a narrative synthesis, presenting thematic summaries of findings [12]. The review protocol (CRD42021260965) was registered on the International Prospective Register of Systematic Reviews (PROSPERO) [13].

### Search strategy and inclusion criteria

The first author (E.N.) conducted a systematic search of bibliographic databases (MEDLINE, CINAHL Plus, PsycINFO and Sociology Database) on 29 November 2021, consulting a subject librarian. We updated the search on 2 February 2023 (Appendix 1, Supplemental Digital Content).

We included articles reporting empirical studies using any qualitative research method, or mixed-methods studies with a substantial qualitative component (extracting only qualitative findings). To be eligible, articles had to be English-language, peer-reviewed, published after 1981, and reporting on people aged ≥16 years with recently acquired HIV (as defined earlier), with their HIV diagnosis having been ≤12 months before recruitment. Grey literature and review articles were excluded.

### Data extraction and management

Potential articles were collated using Covidence. After de-duplication, E.N. screened titles and abstracts. Two independent reviewers (E.N. and S.T.) performed full text screening, reaching consensus on included articles through discussion. E.N., N.P. and S.T. extracted data from the final articles using a standardized form in Covidence, including bibliographical information, methods, patient and public involvement (PPI), setting, population and themes. At least two independent reviewers extracted data.

### Quality appraisal

E.N. and S.T. assessed included articles with the Critical Appraisal Skills Programme (CASP) Qualitative Studies Checklist [14], using it to inform discussions about methodological quality. Articles were classified as ‘weak’, ‘moderate’ or ‘high’ with consensus reached through discussion. We included all articles, regardless of quality assessment.

### Qualitative synthesis

We undertook a textual narrative synthesis of articles [15]. This involved tabulating included articles in a standardized form and summarizing key findings. Summarized findings across articles were organized into themes and compared and contrasted to ascertain commonalities and differences. Themes were then arranged into meta-themes.

### Assessment of confidence in findings

E.N. and S.T. conducted a GRADE-CERQual assessment to determine confidence in the review findings [16]. This involved categorizing confidence in findings as ‘low’, ‘moderate’ or ‘high’ based on methodological limitations, coherence, adequacy, relevance and overall assessment of each review finding.

## Results

### Characteristics of included articles

**Fig. 1:**
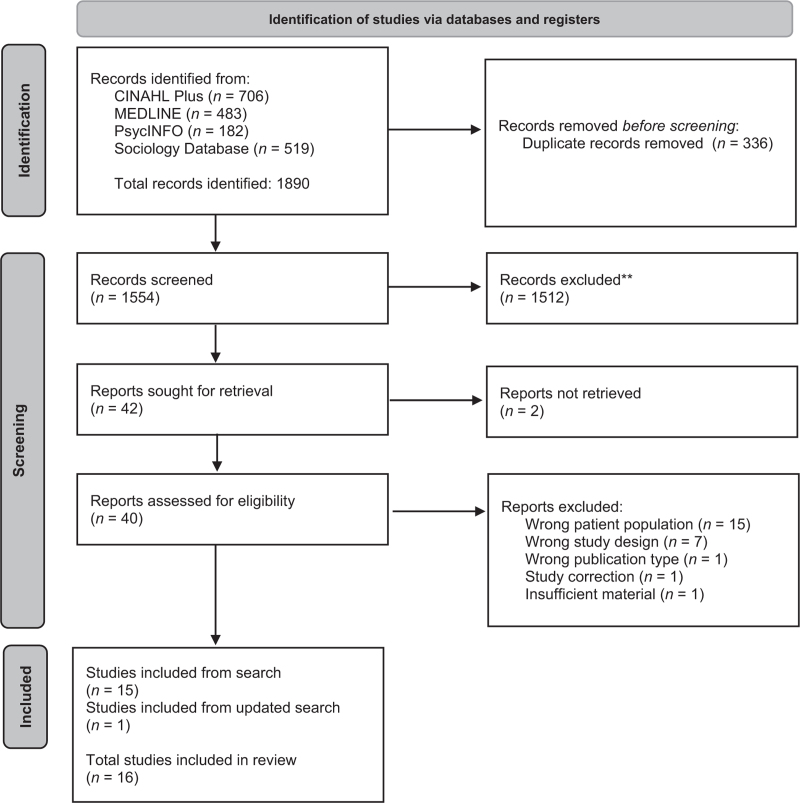
PRISMA diagram.

We reviewed 1890 records, reduced to 1554 following de-duplication (Fig. 1). We excluded 1512 articles at title and abstract stage. We assessed 40 full-text papers, excluding 25 because of participant population (*n* = 15), study design (*n* = 7); publication type (*n* = 2); and lack of sufficient data (*n* = 1).

The remaining 15 articles were included and an additional article was added following the updated search, making the total 16. No additional articles were identified outside these searches. Nine used mixed-methods and seven used semi-structured interviews (SSIs) only. Studies were conducted in Malawi (*n* = 6); South Africa (*n* = 3); USA (*n* = 4); Kenya (*n* = 2); Australia (*n* = 1); Canada (*n* = 1); and England (*n* = 1). Four studies recruited men who have sex with men (MSM), one recruited only women, and two recruited young people (16–26 years) (Table 1). Four articles were from the HPTN 062 study (Malawi) [17–20]; three were from the NIMH Acute HIV Infection Study (USA) [21–23].

**Table 1:** Summary of included studies.

Lead author	Reference	Title	Year of publication	Aim(s)	Country	Study design	Time between diagnosis and data collection	Population	Number of participants	Findings	Involvement of community partners and/or people living with HIV	Quality appraisal
Atkinson	[23]	Psychiatric Context of Acute/Early HIV Infection. The NIMH Multisite Acute HIV Infection Study: IV	2009	To understand the psychiatric context of acute/early HIV infection immediately preceding and following HIV transmission.	USA Pooled samples from selected clinic sites and referral from colleagues^a^	Mixed methods	Interviews conducted at two time periods, within 4 weeks and within 8 weeks of diagnosis	No specific population group (mainly MSM)	34 (82% gay men)	• Participants described managing HIV diagnosis at the same time as pre-existing substance use and mental health conditions as challenging. • Integration into medical and social services and/or communities of people living with HIV was associated with coping.	Not reported	Weak
Corneli	[17]	HPTN 062: A Feasibility and Acceptability Pilot Intervention to Reduce HIV Transmission Risk Behaviors Among Individuals with Acute and Early HIV Infection in Lilongwe, Malawi	2014	To assess the feasibility of MI-based counselling for individuals in the acute and early phases of HIV infection and to evaluate the acceptability of counselling among participants.	Malawi Recruited from single clinic via CHAVI 001	Mixed methods	Interviews at weeks 2 & 8 following diagnosis	No specific population group	27 Brief education arm (*n* = 13); MI intervention arm (*n* = 14)	• The Uphungu Wanga motivational interviewing intervention was found to be feasible and acceptable. • Few major differences found between the arms of the study.	Not reported	Moderate
Corneli	[27]	A descriptive analysis of perceptions of HIV risk and worry about acquiring HIV among FEM-PrEP participants who seroconverted in Bondo, Kenya, and Pretoria, South Africa	2014	To understand HIV risk perceptions and worry about HIV acquisition prior to seroconversion among participants who seroconverted during the FEM-PrEP trial	Kenya & South Africa FEM-PrEP trial	Mixed methods	Interviews at week 1, 4, 8 following diagnosis	Women	51 Pretoria (*n* = 29); Bondo (*n* = 24)	• Four processes of risk nationalization identified: “protective behaviour,” “protective reasoning,” “recognition of vulnerability,” and “no rationalization or action.” • Women who were at substantial risk of acquiring HIV often underestimated this risk • Women who recognized their risk were not always able to act on this.	Not reported	High
Goldbach		What affects timely linkage to HIV care for young men of color who have sex with men? Young men's experiences accessing HIV care after seroconverting	2022	To understand the barriers and facilitators of linkage to care for young MSM of color who are living with HIV	USA Venue-based and social media recruitment	Semi-structured interviews	Within 6 months of seroconversion	MSM of color aged 16–24 years	15	• All but one participant had tested for HIV in the past • Accepting diagnosis tended to follow a grief process and acceptance was important for good linkage to care • Preference for comprehensive services	Not reported	High
Gourlay		A qualitative study exploring the social and environmental context of recently acquired HIV infection among men who have sex with men in South-East England	2017	To explore the social and environmental context in which new HIV infections occurred among MSM in London and Brighton in 2015, understand how these contexts influenced HIV risk and to inform the design of health interventions	England Recruited from UK Register of HIV Seroconverters	Semi-structured interviews	Within 12 months of diagnosis	Gay, bisexual and other MSM	21	• Risk was perceived by participants as multifactorial. • Risk factors included individual psycho-social factors as well as social environment.	Not reported	High
Grace		Diagnostic Technologies in Practice: Gay Men's Narratives of Acute or Recent HIV Infection Diagnosis	2015	To examine new HIV testing technologies in the diagnosis of acute or recent HIV infection in clinical settings.	Canada Recruited from multiple clinics	Semi-structured interviews	Interviews conducted within 14 days of enrollment to study (baseline surveys administered between 8–128 days post-diagnosis)	Gay, bisexual and other MSM	25 Acute diagnosis (*n* = 13); Recently-acquired HIV (*n* = 12)	• New testing technologies have changed the experience of diagnosis and there is some patient and provider uncertainty when it comes to interpreting test results and the meaning of AHI • AHI a strong motivating factor for initiating treatment.	Community partners consulted on development of interview guides.	High
Grodensky		Adaption and delivery of a motivational interviewing-based counseling program for persons acutely infected with HIV in Malawi: Implementation and lessons learned	2018	Exploring the relevance of motivational interviewing with individuals diagnosed with acute HIV infection	Malawi Recruited from single clinic via CHAVI 001	Mixed methods	Weeks 2, 8, 12 & 24 following diagnosis	No specific population group	14 intervention participants (8 men, 6 women); 2 intervention counsellors	• The content of the Uphungu Wanga (MI) intervention was relevant and MI techniques of topic selection and goal setting were viewed positively. • Some elements of the MI intervention did not help participants to explore behaviour change as intended.	Not reported	Weak
Hino		HIV status disclosure during acute HIV infection in Malawi	2018	To explore barriers and facilitators of disclosure to sex partners among individuals diagnosed with AHI and how knowledge of and attitudes toward the person from whom they have acquired HIV affects disclosure	Malawi Recruited from two HIV testing and counselling centres	Semi-structured interviews	4 weeks after enrollment to study	No specific population group	40	• Most participants reported sharing their status with a sexual partner within a month of diagnosis. • Most participants felt they knew, or might know, the person from whom they had acquired HIV.	Not reported	Moderate
Pettifor		Continued High Risk Sexual Behavior Following Diagnosis With Acute HIV Infection In South Africa and Malawi: Implications for Prevention	2011	To understand the sexual behaviours of individuals around the time of diagnosis with AHI	South Africa & Malawi Recruited from clinics in Johannesburg and Lilongwe for CHAVI 001	Mixed methods	Recruited within 13 months of AHI diagnosis (one group newly diagnosed). Interviews conducted at weeks 1, 4 & 12	No specific population group	37 Current AHI (*n* = 19); Previous AHI (*n* = 18) South Africa (*n* = 18); Malawi (*n* = 19)	• Most participants felt they had changed their behaviour since AHI diagnosis, but reported barriers to condom use and abstinence. • Participants expressed a desire for a behavioural intervention at the time of AHI diagnosis.	Not recorded	Moderate
Pettifor		HPTN 062: A Pilot Randomized Controlled Trial Exploring the Effect of a Motivational-Interviewing Intervention on Sexual Behavior among Individuals with Acute HIV Infection in Lilongwe, Malawi	2015	To understand if an MI intervention reduces unprotected sex acts among those with AHI	Malawi Recruited from clinic(s) via CHAVI 001	Mixed methods	Interviews at weeks 2, 8, 12 & 24 after diagnosis	No specific population group	27	• The majority of participants in study reported behaviour change following AHI diagnosis regardless of intervention arm. • Those in the MI arm gave more concrete examples of risk reduction strategies.	Not reported	Moderate
Remien		Lack of Understanding of Acute HIV Infection among Newly-Infected Persons--Implications for Prevention and Public Health: the NIMH Multisute Acute HIV Infection Study: II	2009	To explore awareness and understandings of AHI among people with AHI	USA Pooled samples from selected clinic sites and referral from colleagues^a^	Mixed methods	Interviews conducted at two time periods, within 4 weeks and within 8 weeks of diagnosis	No specific population group (mostly MSM)	34 (82% gay men)	• Lack of awareness of AHI symptoms and AHI testing methods • Little understanding of meaning of AHI and implications for infectiousness.	Not reported	High
Ritchwood		Understanding of perceived infectiousness and its influence on sexual behavior among individuals with acute HIV infection in Lilongwe, Malawi (HPTN062)	2020	To assess understanding of the association between AHI and viral load, and its connection to sexual behavior among participants in a MI intervention study	Malawi Recruited from single clinic via CHAVI 001	Mixed methods	Interview conducted 8 weeks following diagnosis	No specific population group	26 (9 women)	• Most participants understood key aspects of AHI, but those receiving the MI intervention gave more detailed descriptions of their understanding. • Nearly all participants understood that they were highly infectious.	Not reported	Weak
Slavin		Understandings of risk among HIV seroconverters in Sydney	2004	To identify the event which resulted in HIV infection; to explore perceptions of risk in relation to the sexual practices involved in that event; to explore the meanings the participant attaches to the event	Australia Recruited through a network of general practitioners and specialists	Semi-structured interviews	Interviews two weeks to several months after diagnosis. Conducted between July 1993 and March 2001	Gay, bisexual and other MSM	92	• ‘Rational/objectivist’ discourse of risk sees risk as knowable through the use of scientific knowledge. Risk may be calculated. • ‘Social/subjectivist’ discourse of risk sees risk as socially and culturally embedded	Not reported	High
Steward		Behavior Change Following Diagnosis with Acute/Early HIV Infection--A Move to Serosorting with Other HIV-Infected Individuals. The NIMH Acute HIV Infection Study: III	2009	To examine changes in sexual risk behaviours in those with acute/early HIV.	USA Pooled samples from selected clinic sites and referral from colleagues^a^	Mixed methods	Interviews conducted at two time periods, within 4 weeks and within 8 weeks of diagnosis	No specific population group (mainly MSM	28 (26 MSM)	• Changes in sexual behavior to reduce risk motivated by concerns about passing HIV on to others.	Not reported	Moderate
Van Der Elst		Adjustment to acute or early HIV-1 infection diagnosis to prompt linkage to care and ART initiation: qualitative insights from coastal Kenya	2019	To understand factors that facilitate adjustment to AHI/early HIV diagnosis, and factors which enable or obstruct initiation of ART and risk reduction recommendations	Kenya Recruited from multiple clinics; and also an on-going ‘at-risk’ male sex worker cohort	Semi-structured interviews	Two interviews conducted: within two weeks of diagnosis, and three months following diagnosis	No specific population group	17 (9 women)	• Identified three important needs for people with AHI and early infection: (1) to better understand and accept status; (2) to develop strategies to adjust to the reality of status; (3) to protect self and others.	Not reported	Moderate
Wolpaw		Patient Experiences following Acute HIV Infection Diagnosis and Counseling in South Africa	2014	To understand how a diagnosis of AHI interpreted and understood To investigate the challenges of AHI diagnosis in resource poor areas.	South Africa Recruited from single clinic	Semi-structured interviews	Interview conducted one week after diagnosis	Young people (aged 18–26)	6	• Following counselling, many participants accepted the testing regimen used to diagnose AHI and used the recency of infection to identify the person from whom they acquired HIV. • Many did not retain or find important information given regarding increased infectiousness.	Not reported	Weak

a Not described in article, see Kerndt *et al.* (2009).

All articles were published in 2000 or later. One included interviews conducted in 1993–2001 [24], meaning all articles covered the period after introduction of effective treatment, with one also including interview material from before this time.

**Fig. 2:**
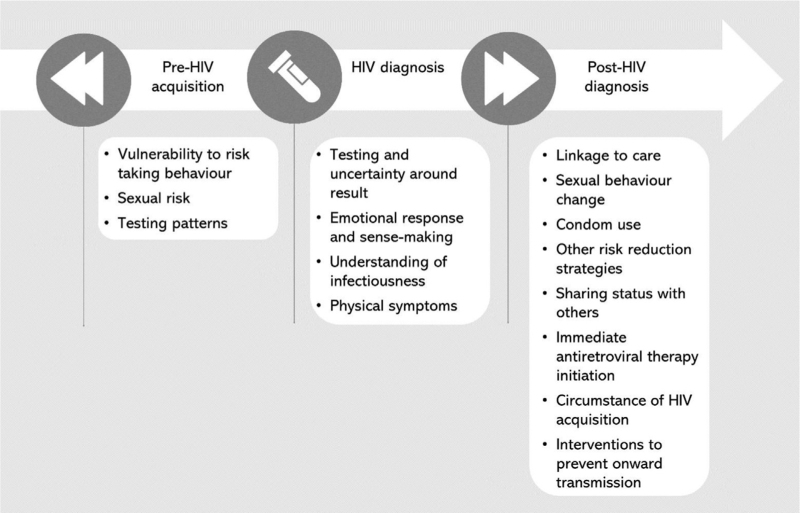
Themes.

We identified 15 themes, structured in chronological time from pre-HIV acquisition, to HIV diagnosis, through to post-HIV diagnosis (Figure 2).

### Pre-HIV acquisition

Themes pertaining to the time before HIV acquisition focus primarily on sexual risk, including circumstances contributing to vulnerability to risk-taking behaviour, or how risk is managed and rationalized in sexual encounters. Testing patterns prior to HIV diagnosis are also included here.

#### Vulnerability to risk-taking behaviour

The two studies exploring vulnerability to risk-taking behaviour focused on MSM (one included only MSM participants [25]; and 30 of 34 participants in the other were MSM [23] (reported separately [26])). This was primarily driven by psychosocial factors, including life stressors such as financial insecurity and work [25]; mental health issues including depression [23,25], feelings of low self-worth, loss of purpose, and previous psychological trauma [25]. Other factors included drug and alcohol use [23,25].

Close proximity and access to nightclubs or sex-on-premises venues offered increased risk-taking opportunities, with around half of participants in one study describing sexualized drug use and several participants commenting on the ‘normalization’ of injecting [25]. Community perceptions of treatment advances in HIV were also reported as leading to complacency, resulting in increased sexual risk-taking [25].

#### Sexual risk

Two studies asked participants about sexual risk prior to diagnosis of recently acquired HIV. One, in women, described “processes of risk rationalization” [27]. This included ‘protective behaviour’, that is, using one or more strategy of risk reduction (most commonly condoms or having only one sexual partner). Another process was “protective reasoning” meaning assuring themselves they were at low risk of HIV acquisition due to previous HIV-negative tests, or believing that a relationship was monogamous. Some acknowledged their HIV risk, that is, due to male partners’ preference for condomless sex, cultural traditions or gender roles limiting autonomy in sexual decision-making, but were unable to consistently address risk behaviour. In contrast, research from Australia found that MSM reported calculated risk-taking, considering information such as a sexual partner's HIV viral load, and prioritizing passion and intimacy [24]. This study also described choosing *not* to use condoms to avoid ‘othering’ partners who they knew were living with HIV [24].

PrEP was only mentioned once; MSM in a 2015 UK study (predating widespread availability of PrEP in the UK) described unsuccessful attempts to access PrEP [25].

#### Testing patterns

One US study of MSM of colour aged 16–24 reported all but one participant having tested for HIV prior to diagnosis. Those who tested frequently generally reported that this was motivated by a desire to make “good choices” or ‘need[ing] to know’. Social and support networks were also facilitators for testing, including through encouragement or being accompanied by friends when testing [28].

### HIV diagnosis

A diagnosis of recently acquired HIV may be complicated by acute symptoms and indeterminate HIV tests. It is also a time of high viraemia and risk of transmission. This meta-theme highlights diagnosis as an emotive time, sometimes accompanied by anxiety or confusion generated by negative or indeterminate test results.

#### Testing and uncertainty around result

Diagnosis during AHI can be complicated by negative or indeterminate HIV antibody test results leading to confusion [22,29], mistrust of the diagnosis [29], and anxiety precipitated by healthcare providers’ (HCP) own uncertainty [30]. In one study, some participants accessed confirmatory testing soon after diagnosis, while others first processed fears and concerns [28]. Those with stronger relationships with HCPs were more likely to seek confirmatory testing [28].

#### Emotional response and sense-making

Emotional responses to diagnosis included shock, anger, depression, fears of dying [29], disbelief and denial [31]. One study described participants being “engulfed” in thinking about diagnosis, and acceptance akin to a grief process [28]. Adjusting to diagnosis was particularly challenging for those who had pre-existing mental health or substance issues [23], but was facilitated by engagement with health and social care, and with communities of people living with HIV. It is unclear whether these experiences were specific to recently acquired HIV. Indeed, one study found that participants (all MSM) did not, on the whole, feel that their diagnosis of AHI impacted their understanding of themselves as living with HIV [30].

#### Understanding of infectiousness

Studies reported limited understanding among participants of increased infectiousness during AHI [29,32]. Some had been informed of the increased risk of onward transmission, but had struggled to retain this information [22,29], largely as a result of the emotional impact of diagnosis [31]. Two articles – one from Canada with MSM, and another reporting on an intervention trial in Malawi – described a good understanding that increased infectiousness resulted from a high viral load [20,30]. Another study, from the USA, reported that understanding and awareness were more prominent among MSM who were highly-educated and well integrated into urban gay communities [22].

#### Physical symptoms

Two studies explored AHI symptoms. One, predominantly with MSM, reported that the majority of participants described symptoms consistent with AHI including flu-like illness, headaches, rashes and lymphadenopathy [22]. These were rarely recognized as potentially indicative of AHI; those who were aware that their symptoms could suggest AHI had adapted their sexual behaviour to reduce risk of transmission [22]. Several interviewees indicated that they would have sought testing earlier had they recognized these symptoms, and would have valued the opportunity to prevent onward transmission [22]. Another study among MSM found that knowledge of AHI and natural history of HIV infection facilitated coping through understanding of AHI symptoms as transient [30].

### Post-HIV diagnosis

The time post-diagnosis is our most substantial meta-theme, predominantly focusing on sexual behaviour and risk reduction post-diagnosis. We also include experiences of immediate ART initiation and sharing HIV status with others, as well as identifying circumstances of HIV acquisition.

#### Linkage to care

One study found that young MSM of colour preferred comprehensive services which would address their needs without onward referral. HIV specialist care was favoured as it was perceived to be welcoming, caring and ‘gay friendly’ [28].

#### Sexual behaviour change

Sexual behaviour change following diagnosis was discussed in seven articles [18,21,23,29–32]. Some reported sexual risk reduction to prevent onward transmission, while others demonstrated no behaviour modification as participants adjusted to their diagnosis.

Reducing alcohol intake was identified by some participants as an important way of reducing sexual risk [18]. Sexualized drug use was rarely explored; one study on the mental health aspects of early HIV highlighted potential anxiety about sex without the influence of drugs [23].

#### Condom use

Five studies explored condom use following diagnosis [18,21,29,31,32]. The majority were conducted in African countries (South Africa, Malawi and Kenya) with one from the USA (almost all participants were MSM). Most reported increased condom use or intention to use condoms. Motivations included preventing transmission, avoiding the need to share one's status with a partner [32], and avoiding re-infection with another HIV sub-type [18,32]. A trial of an AHI health promotion intervention in South Africa found that participants reported planning to use condoms with future partners [29].

Other studies reported barriers to condom use including partner type (finding it harder to introduce condoms with primary partners), partner refusal [18,32], a partner already living with HIV (or assumption they were living with HIV), uncertainty about condom efficacy, wanting to conceive [32], lack of confidence in condom use [18] and concerns about sexual pleasure [18,21].

#### Other risk reduction strategies

Other risk-reduction strategies following diagnosis of recently acquired HIV included limiting the number of sexual partners [18,32], serosorting (seeking partners also living with HIV) [21], or sexual abstinence [18,21,30–32]. Abstinence was often a temporary measure during AHI [18,30]. Reasons included transmission concerns [21,30,32], fears of re-infection [32], illness and distress [21], loss of libido [21,32], and lack of opportunity [18,32]. Abstinence was particularly difficult for those who were married [18].

#### Sharing status with others

Four articles explored how HIV status was discussed with others. One study, conducted in the US with young MSM of colour found that sharing status (also known as disclosing) with others was important but not integral to adapting to diagnosis [28].

Three studies (Malawi and South Africa) [18,32,33], focused on sharing status with a partner. This was a key facilitator of sexual behaviour change, enabling condom use [18,32]. However, telling all sexual partners was rare [33]. In one study, most participants who shared their HIV status with a partner did so soon after their AHI diagnosis; most who had not yet shared their status planned to test together as a couple [33]. Drivers of telling partners were emotional investment in the relationship and a belief that their partner should know [33]. Relationship type shaped decision-making, with participants less likely to share their status with a casual partner [33]. Reasons for not sharing one's status included fear of partner reaction (including rejection) [33].

#### Immediate ART initiation

Prompt initiation of ART during AHI optimizes health and reduces the risk of onward transmission. Two studies, one in MSM (Canada) [30], and one in people attending HIV clinics including male sex workers (Kenya) [31] explored immediate ART in AHI. It was found to be acceptable, with participants describing starting ART as “rewarding” in that it improved acute symptoms [31] and reduced HIV viral load [30]. Early ART initiation was often encouraged by HCPs, and participants were confident that ART would restore and maintain health [31], and reduce the risk of onward sexual transmission [30]. For cisgender women with AHI, immediate ART allowed them to fulfil their maternal role either by remaining healthy to care for children, or preventing vertical HIV transmission [31].

#### Circumstance of HIV acquisition

Recency of infection allowed participants to identify how and when they were likely to have acquired HIV [25,29,30,33]. Those with multiple sexual partners used this knowledge to narrow down the person from whom they were most likely to have acquired HIV [29]. Time between a sexual encounter and symptoms served the same purpose [33].

Being monogamous, having had negative HIV tests prior to having sex with a partner, or having condomless sex were other reasons participants felt they could identify the person from whom they had acquired HIV [33]. Rumours about the HIV status of previous partners, HIV status of partners of partners, or health of previous partners (e.g. weight loss) were also cited as potential indicators [33]. In one study, participants largely believed they had acquired HIV at sex-on-premises venues, parties, or through sex with a casual partner they met online; a minority thought they had acquired HIV from a regular partner, or as a result of sexual coercion [25].

One study (Malawi) focused on sharing HIV status with the person from whom HIV acquisition was suspected [33]. Participants who reported knowing who this was (often a spouse or primary partner) stated that they shared their status with them soon after diagnosis. Some thought they had been knowingly exposed to HIV and felt deceived and angry. Participants who felt their partners were not aware of their own HIV status were sometimes reluctant to share their diagnosis for fear of being blamed for transmission.

#### Interventions to prevent onward transmission

One article reported research informing the development of prevention strategies during AHI [32]; interventions were viewed favourably by participants and thought to be best initiated within a week of diagnosis [32]. Four articles reported results from HPTN 062, a motivational interviewing (MI) intervention study in Malawi [17–20]. MI starting on or soon after AHI diagnosis was reported to be feasible and acceptable [17]. It was found to provide information on AHI and transmission, behavioural guidance, and support in coping with diagnosis [17] and to facilitate sexual behaviour change [18]. Advice about having children whilst living with HIV was seen as particularly helpful [19].

## Discussion

Our narrative textual synthesis identified 15 themes, organized chronologically from pre-HIV acquisition, to HIV diagnosis, through to post-HIV diagnosis. Many of our findings are not specific to recently acquired HIV. A recent systematic review of qualitative studies on experiences and attitudes of people living with HIV highlights the negative psychological impact of HIV diagnosis and the role of information in coping and self-management [34].

Our review differs from previous qualitative work on HIV diagnosis by focusing specifically on recently acquired HIV. This generates unique insights. Overall, understanding of recently acquired HIV, and specifically AHI, varied across studies and populations. Three themes were graded as high confidence, indicating that they are likely to represent the views of people with recently acquired HIV (Table 2). First, indeterminate HIV test results in AHI generate uncertainty and impact psychological wellbeing. This highlights the importance of multidisciplinary support (including psychology and peer support) in helping people manage emotional consequences. Second, diagnosis of recently acquired HIV (especially AHI) is a powerful lever for sexual risk reduction, mainly through abstinence. This emphasizes the role of health promotion to reduce the risk of onward transmission during this critical time. However, there must also be an acknowledgement of the potential negative impacts on future sexual wellbeing. The onus is on HCPs to revisit discussions about condom use and sexual abstinence once people are virologically suppressed on ART. Finally, qualitative research with people with recently acquired HIV provides unique insights into the complexity of circumstances around HIV acquisition. New HIV infections continue to occur globally, despite TasP and PrEP. Continued investment in quantitative *and* qualitative research on recently acquired HIV is essential if we are to understand why infections occur and how we can optimize prevention strategies.

**Table 2:** GRADE-CERQual assessment.

Summary of review finding	Studies contributing to the review finding	Assessment of methodological limitations	Assessment of coherence	Assessment of adequacy	Assessment of relevance	Overall CERQual assessment of confidence in the evidence	Explanation of CERQual assessment
Pre-HIV acquisition							
Vulnerability to risk taking behavior	[23,25]	No concerns: [25] Serious concerns: [23]	Minor concerns: both studies clearly highlight the relationship between vulnerability to risk taking behavior and HIV acquisition.	Serious concerns: only two studies contribute data supporting this theme.	Moderate concerns: these findings may apply to all people diagnosed with HIV and not just those with recently acquired infection.	Low confidence	This was graded as low confidence. We have minor concerns about coherence, however, only two studies contribute to this theme (one of which has serious methodological limitations). Furthermore, these findings are unlikely to be specific to recently acquired HIV.
Sexual risk	[24,25,27]	No concerns: [24,25,27]	Moderate concerns: all three studies contributing to this theme have different focus.	Moderate concerns: only three studies contribute to this theme. However, the data included are rich and of a high quality.	Minor concerns: although sexual risk taking applies regardless of the timing of infection, the fact the participants were interviewed close to the time of acquisition means they are more likely to recall risk taking.	Moderate confidence	This was graded as moderate confidence. Only three studies contribute to this theme, however they are methodologically robust and the data are rich and relevant.
Testing patterns	[28]	No concerns: [28]	Serious concerns: there is only one study and limited data are presented.	Serious concerns: only one study contributing to this theme.	Moderate concerns: these findings may apply to all people diagnosed with HIV and not just those with recently acquired infection.	Low confidence	This was graded as low confidence. There was only one study contributing to this finding, which was of high quality, however testing was not the focus.
HIV diagnosis							
Testing and uncertainty around result	[22,28–30]	No concerns: [22,28,30] Serious concerns: [29]	Minor concerns: three studies describe the confusion and uncertainty that arises from negative or indeterminate test results during AHI. The fourth study discussed the importance of relationships with healthcare providers in seeking confirmatory testing.	Moderate concerns: only three studies contribute to the key finding around uncertainty.	No concerns: the uncertainty described is unique to AHI.	High confidence	This was graded as high confidence. Three of the four studies are of high quality. The finding is coherent and relevant.
Emotional response and sense-making	[23,28–31]	No concerns: [28,30] Moderate concerns: [31] Serious concerns: [23,29]	No concerns: across all studies there is a compelling narrative of the emotional consequences of HIV diagnosis.	No concerns: five studies contribute to this finding.	Moderate concerns: these findings may apply to all people diagnosed with HIV and not just those with recently acquired infection.	Moderate confidence	This was graded as moderate confidence. Five studies contribute to this theme however the finding is unlikely to be specific to a diagnosis of recently acquired HIV.
Understanding of infectiousness	[20,22,29–32]	No concerns: [22,30] Moderate concerns [31,32] Serious concerns: [20,29]	Moderate concerns: the six studies present mixed results. four studies report limited understanding, sometimes as a result of difficulty retaining information. Whereas two studies reported good understandings.	Moderate concerns: six studies contribute to this theme, however the majority lack in depth reporting of empirical qualitative findings.	No concerns: this finding is specific to recently acquired HIV.	Moderate confidence	This was graded was graded as moderate confidence. Six studies contribute to this theme, however findings are mixed and some studies lack depth. The findings are highly specific to recently acquired HIV.
Physical symptoms	[22,30]	No concerns: [22,30]	Serious concerns: only two studies contribute to this finding and they explore different aspects of symptoms.	Serious concerns: only two studies contribute to this finding.	No concerns: this finding is specific to recently acquired HIV.	Low confidence	This was graded as low confidence despite the inclusion of two high quality studies. This is because there are only two studies contributing and they both report on different aspects of symptoms.
Post-HIV diagnosis							
Linkage to care	[28]	No concerns: [28]	Serious concerns: the finding is coherent, however it is not compelling due to there only being one study contributing to this finding.	Serious concerns: only one study contributes to this finding.	Moderate concerns: these findings may apply to all people newly diagnosed with HIV and the authors do not specifically draw out how recent acquisition of HIV impacts linkage to care.	Low confidence	This has been graded as low confidence due to there only being one study contributing and a lack of engagement with recently acquired specifically.
Sexual behavior change	[18,21,23,29–32]	No concerns: [30] Moderate concerns: [18,21,31,32] Serious concerns: [23,29]	Moderate concerns: findings are mixed across the seven studies that contribute to this theme. Some studies report behavior modification whilst others report no change.	No concerns: seven studies contribute to this finding.	Moderate concerns: these findings may apply to all people newly diagnosed with HIV	Low confidence	This has been graded as low confidence as study findings are mixed and do not specifically relate to recently acquired HIV.
Condom use	[18,21,29,31,32]	Moderate concerns: [18,21,31,32] Serious concerns: [29]	Minor concerns: five studies contribute to this finding. The majority report increased use or intention to use condoms. Studies report various barriers to condom use.	Minor concerns: five studies contribute to this finding, ranging from moderate to low quality.	Moderate concerns: these findings may apply to all people living with HIV and the authors do not specifically draw out how recent acquisition of HIV impacts condom use.	Moderate confidence	This has been graded as moderate confidence as five studies contribute to this finding and are consistent. However, the finding may not be specific to recently acquired HIV.
Other risk reduction strategies	[18,21,30–32]	No concerns: [30] Moderate concerns: [18,21,31,32]	Minor concerns: five studies contribute to this finding and all report sexual abstinence as a risk reduction strategy. Studies report various other strategies in addition.	No concerns: five studies contribute to this finding.	Minor concerns: several studies specifically comment on abstinence in the context of recently acquired HIV.	High confidence	This has been graded as high confidence as five studies contribute and study findings are consistent and relate to recently acquired HIV.
Sharing status with others	[18,28,32,33]	No concerns: [28] Moderate concerns: [18,32,33]	Serious concerns: four studies contribute to this finding. However, these studies differ in their focus and their conclusions.	Moderate concerns: the majority of findings come from one study which is of moderate quality.	Moderate concerns: these findings may apply to all people living with HIV and not just those with recently acquired infection.	Low confidence	This has been graded as low confidence as study findings are mixed and do not specifically relate to recently acquired HIV.
Immediate antiretroviral therapy initiation	[30,31]	No concerns: [30] Moderate concerns: [31]	Minor concerns: only two studies contribute to this finding, however they are consistent in their conclusions.	Serious concerns: only two studies contribute to this finding.	No concerns: this finding is highly relevant to recently acquired HIV.	Moderate confidence	This has been graded as moderate confidence. Although only two studies contribute, they are consistent in their conclusions and are specific to recently acquired HIV.
Circumstance of HIV acquisition	[25,29,30,33]	No concerns: [25,30] Moderate concerns: [33] Serious concerns: [29]	Minor concerns: four studies contribute to this finding and they are consistent in their conclusions.	Minor concerns: four studies contribute to this finding, two of which are of a high quality.	No concerns: this finding is highly relevant to recently acquired HIV.	High confidence	This has been graded as high confidence. Four studies contribute to this finding which is highly specific to recently acquired HIV.
Interventions to prevent onward transmission	[17–20,32]	Moderate concerns: [17,18,32] Serious concerns: [19,20]	Serious concerns: all five studies relate to the same intervention and it is therefore hard to assess coherence of findings.	Serious concerns: all five studies relate to the same intervention. This limits conclusions about prevention interventions for people with recently acquired HIV.	No concerns: this is highly specific to recently acquired HIV.	Low confidence	This has been graded as low confidence due to all included studies reporting on the same intervention in the same population.

There are important gaps in existing literature. Even in more recent years, we found no qualitative studies exploring the impact of PrEP and/or TasP on sexual risk in the context of recently acquired HIV. There is an absence of research investigating the use (or non-use) of PrEP amongst those with recently acquired HIV, particularly access and adherence. There was also no in-depth exploration of the role of drug use (including sexualized drug use) in recent acquisition of HIV. These data are vital if we are to identify missed opportunities for HIV prevention. Furthermore, it is important to understand how PrEP and U=U impact stigma experienced by those with recently acquired HIV.

The vast majority of studies describe circumstances of HIV infection. Risk is framed primarily in terms of individual psychological or behavioural factors, often failing to situate risk in the broader context of people's lives. This narrow focus on HIV acquisition neglects other important aspects of lived experiences such as immediate ART, stigma, and impacts on broader health and wellbeing. Studies in research-rich settings tend to focus on MSM populations, while studies including women in resource-limited settings fail to disaggregate data, hindering our understanding of recently acquired HIV in cisgender women. Key populations including transgender and non-binary people and people from racially-minoritized communities, are under-represented.

Only six of the included articles were assessed as high quality. A limitation across the literature was that engagement with the qualitative data was often superficial and lacked ‘thick’ description [35], precluding an in-depth textual line-by-line analysis of empirical data in this review. Most work was not theoretically-informed and lacked rigour, and positionality of the researcher(s) was rarely considered. Additionally, no articles reported that people living with HIV had been involved in study design, conduct or analysis; only one reported consulting with community partners on study design. The involvement of people with lived experience would strengthen the quality of this work, and ensure that the needs and priorities of people living with HIV are taken into account [36].

### Strengths and limitations of the review

We conducted a systematic and comprehensive search of the literature and completed standardized data extraction. Full text review, extraction, and quality and confidence assessment were conducted by two independent reviewers.

The review is limited by the exclusion of grey literature and studies not published in English.

### Conclusion

This is the first systematic review to synthesize qualitative literature on recently acquired HIV. It highlights the importance of support and health promotion at the time of diagnosis, which may facilitate uptake of ART and reduce sexual risk, as well as helping people cope with the uncertainty generated by indeterminate HIV test results. We lack qualitative data on missed opportunities of PrEP and U=U, and how they shape experiences of recently acquired HIV in the contemporary era. This understanding is crucial if we are to achieve the goal of ending HIV by 2030, and to ensure that those with recently acquired HIV are supported holistically to optimize their long-term health and wellbeing.

## Acknowledgements

We acknowledge the invaluable role of Chris Sandford (CASCADE Community Representative) who sadly passed away in November 2022.

Author contributions: Conceptualization: E.N., S.T., F.B.; Formal Analysis: E.N., N.P., S.T.; Writing - original draft: E.N.; Writing - review & editing: E.N., N.P., A.V., B.S., F.B., E.R., S.T.; Supervision: S.T.

CASCADE Collaboration: CASCADE Executive Committee: Santiago Moreno (Chair), Fiona Burns, Rafael Eduardo Campo, Harmony Garges, Cristina Mussini, Nikos Pantazis, Barbara Pinto, Kholoud Porter, Caroline Sabin, Shema Tariq, Giota Touloumi, Vani Vannappagari, Alain Volny Anne, Lital Young

CASCADE Scientific Steering Committee: John Gill (co-chair), Kholoud Porter (co-chair), Christina Carlander, Rafael Eduardo Campo, Harmony Garges, Sophie Grabar, Inma Jarrín, Laurence Meyer, Barbara Pinto, Giota Touloumi, Marc van der Valk, Vani Vannappagari, Alain Volny Anne, Linda Wittkop, Lital Young

CASCADE Social Science Sub-Committee: Shema Tariq (Chair), Agnes Aisam, Diana Barger, Udi Davidovich, Marie Dos Santos, Lars Eriksson, Eli Fitzgerald, John Gill, Sophie Grabar, Inma Jarrín, Argyro Karakosta, Hartmut Krentz, Cristina Mussini, Emily Jay Nicholls, Nicoletta Policek, Elisa Ruiz-Burga, Chris Sandford, Bruno Spire, Inés Suárez-García, Giota Touloumi, Alain Volny Anne

### Conflicts of interest

This research is funded by ViiV Healthcare UK, Janssen Pharmaceutica NV, and Merck Sharp & Dohme Corporation. The study funders had no role in study design, data collection, data analysis, data interpretation, or writing of the report.

S.T. has received speaker honoraria and consultancy fees from Gilead Sciences and speaker honoraria from ViiV Healthcare. F.B. has received funding from Gilead Sciences Ltd for preparation and delivery of educational materials. For the remaining authors none were declared.

## Supplementary Material

Supplemental Digital Content
