# Supplementary material for: A systematic review of qualitative research on recently acquired HIV
Source: AIDS. 2023 Aug 23;37(14):2199–212. doi: 10.1097/QAD.0000000000003697 (PMC10621639; doi:10.1097/QAD.0000000000003697)
Supplement: Supplemental Digital Content [file aids-37-2199-s001.doc]

## **Appendix 1**

**Search strategy**

1 HIV infections/ OR HIV seropositivity/

2 HIV OR Human Immunodeficiency Virus OR HTLVIII OR HTLV III OR HTLV3

**AND**

3 seroconver* OR sero-conver* OR recently infected OR recently acquired OR newly infected OR newly acquired OR recent HIV infection OR acute HIV OR primary HIV OR acute infection

**AND**

4 Interview* OR focus group* OR lived experience OR qualitative OR ethnograph* OR thematic OR diary OR visual methods OR behavior change OR behavior change OR Photovoice

**Search terms**

**MEDLINE (Ovid):**

ab=abstract

kf=keyword heading word

ti=title

HIV.ab.kf.ti

Human Immunodeficiency Virus.ab.kf.ti

HTLVIII.ab.kf.ti

HTLV III.ab.kf.ti

HTLV3.ab.kf.ti

seroconver*.ab.kf.ti

sero-conver*.ab.kf.ti

recently infected.ab.kf.ti

recently acquired.ab.kf.ti

newly infected.ab.kf.ti

newly acquired.ab.kf.ti

recent HIV infection.ab.kf.ti

acute HIV.ab.kf.ti

primary HIV.ab.kf.ti

acute infection.ab.kf.ti

Interview*.ab.kf.ti

focus group*.ab.kf.ti

lived experience.ab.kf.ti

qualitative.ab.kf.ti

ethnograph*.ab.kf.ti

thematic.ab.kf.ti

diary.ab.kf.ti

visual methods.ab.kf.ti

behaviorchange.ab.kf.ti

behaviorchange.ab.kf.ti

Photovoice.ab.kf.ti

**CINAHL Plus (EBSCOhost):**

/=MeSH terms

ab=abstract

ti=title

HIV seropositivity/

HIV Infections/

HIV.ab.ti

Human Immunodeficiency Virus.ab.ti

HTLVIII.ab.ti

HTLV III.ab.ti

HTLV3.ab.ti

seroconver*.ab.ti

sero-conver*.ab.ti

recently infected.ab.ti

recently acquired.ab.ti

newly infected.ab.ti

newly acquired.ab.ti

recent HIV infection.ab.ti

acute HIV.ab.ti

primary HIV.ab.ti

acute infection.ab.ti

Interview*.ab.ti

focus group*.ab.ti

lived experience.ab.ti

qualitative.ab.ti

ethnograph*.ab.ti

thematic.ab.ti

diary.ab.ti

visual methods.ab.ti

behaviorchange.ab.ti

behaviorchange.ab.ti

Photovoice.ab.ti

**PsycINFO (Ovid):**

ab=abstract

id=key concepts

ti=title

HIV.ab.id.ti

Human Immunodeficiency Virus.ab.id.ti

HTLVIII.ab.id.ti

HTLV III.ab.id.ti

HTLV3.ab.id.ti

seroconver*.ab.id.ti

sero-conver*.ab.id.ti

recently infected.ab.id.ti

recently acquired.ab.id.ti

newly infected.ab.id.ti

newly acquired.ab.id.ti

recent HIV infection.ab.id.ti

acute HIV.ab.id.ti

primary HIV.ab.id.ti

acute infection.ab.id.ti

Interview*.ab.id.ti

focus group*.ab.id.ti

lived experience.ab.id.ti

qualitative.ab.id.ti

ethnograph*.ab.id.ti

thematic.ab.id.ti

diary.ab.id.ti

visual methods.ab.id.ti

behaviorchange.ab.id.ti

behaviorchange.ab.id.ti

Photovoice.ab.id.ti

**Sociology Database (ProQuest):**

ab=abstract

ti=title

HIV.ab.ti

Human Immunodeficiency Virus.ab.ti

HTLVIII.ab.ti

HTLV III.ab.ti

HTLV3.ab.ti

seroconver*.ab.ti

sero-conver*.ab.ti

recently infected.ab.ti

recently acquired.ab.ti

newly infected.ab.ti

newly acquired.ab.ti

recent HIV infection.ab.ti

acute HIV.ab.ti

primary HIV.ab.ti

acute infection.ab.ti

Interview*.ab.ti

focus group*.ab.ti

lived experience.ab.ti

qualitative.ab.ti

ethnograph*.ab.ti

thematic.ab.ti

diary.ab.ti

visual methods.ab.ti

behaviorchange.ab.ti

behaviorchange.ab.ti

Photovoice.ab.ti
